# DNA methylation profiles capturing breast cancer heterogeneity

**DOI:** 10.1186/s12864-019-6142-y

**Published:** 2019-11-07

**Authors:** Xiao Chen, Jianying Zhang, Xiaofeng Dai

**Affiliations:** 1 School of Biotechnology, Jiangnan University, Wuxi, Jiangsu China; 2 Henan Academy of Medical and Pharmaceutical Sciences, Zhengzhou University, Zhengzhou, China; 3 Wuxi School of Medicine, Jiangnan University, Wuxi, Jiangsu China

## Abstract

**Background:**

As one of the most described epigenetic marks in human cancers, DNA methylation plays essential roles in gene expression regulation and has been implicated in the prognosis and therapeutics of many cancers. We are motivated in this study to explore DNA methylation profiles capturing breast cancer heterogeneity to improve breast cancer prognosis at the epigenetic level.

**Results:**

Through comparisons on differentially methylated CpG sites among breast cancer subtypes followed by a sequential validation and functional studies using computational approaches, we propose 313 CpG, corresponding to 191 genes, whose methylation pattern identifies the triple negative breast cancer subtype, and report cell migration as represented by extracellular matrix organization and cell proliferation as mediated via MAPK and Wnt signalings are the primary factors driving breast cancer subtyping.

**Conclusions:**

Our study offers novel CpGs and gene methylation patterns with translational potential on triple negative breast cancer prognosis, as well as fresh insights from the epigenetic level on breast cancer heterogeneity.

## Background

Breast cancers are highly heterogeneous, which can at least be classified into luminal, HER2 positive (HER2p) and triple negative (TN) types of tumors given their intrinsic differences in the transcriptional expression pattern and clinical outcome association [1, 2]. TN breast cancers are malignant, lack effective targeted therapy, and is not homogeneous that complicates its diagnosis and therapeutics. DNA methylation plays essential roles in numerous cellular processes such as embryonic development, genomic imprinting, cell differentiation and senescence, deregulation of which contributes to several human diseases including cancers [2]. DNA methylation markers are more chemically and biologically stable than RNA and most proteins [3], thus have emerged as an important class of diagnostic or prognostic markers [4–6], with some of which already being applied in clinics [3]. In this regard, several computational approaches have been established to model methylation patterns including, e.g., Bayesian network that has been applied to analyze chromatin interactions [7]. Here, we are motivated to identify the primary DNA methylation profiles that captures the heterogeneity of breast cancers and can be used to distinguish TN tumors from the rest breast cancer subtypes, with the aim of identifying epigenetic marks or targets facilitating the diagnosis and therapeutics of TNBCs and gaining insights on the epigenetic drivers differentiating breast cancer subtypes.

## Results

### Primary methylation profiles differentiating breast cancer subtypes

**Fig. 1:**
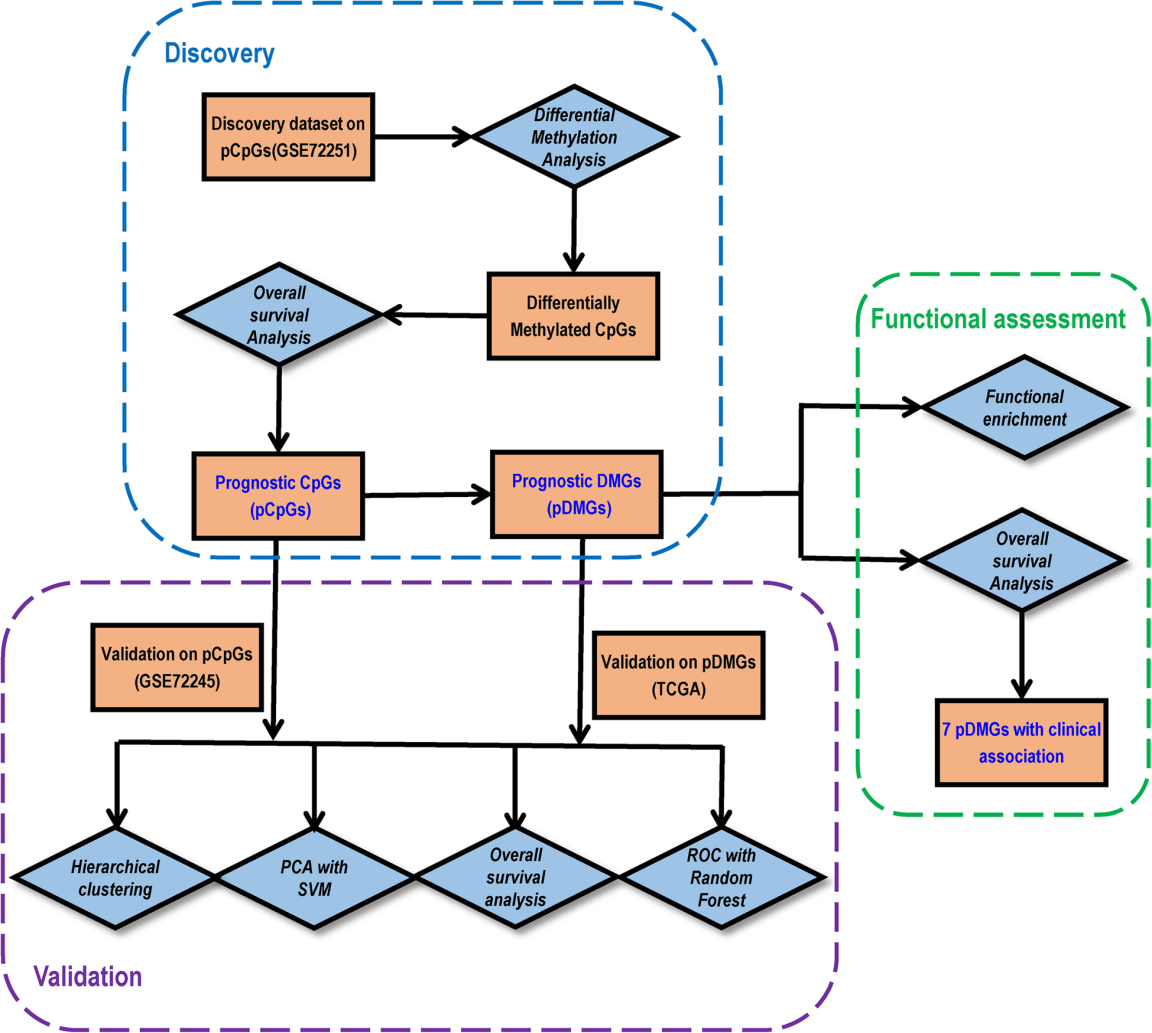
Study workflow. Blue diamond represents analysis steps and orange square shows data or results

The overall methylation profile of each breast cancer subtype, computed as the average of all DNA methylation sites in each subtype, showed that DNA methylation status decreases in the order of HER2p, luminal, and TN subtypes, and the methylation pattern of luminal cancers is more dispersedly distributed than that of the other subtypes (as the heterogeneity of luminal cancers is higher than the other subtypes that can be further divided into the luminal A and B subtypes) (Fig. 1a). The difference on overall methylation patterns across breast cancer subtypes does not reach statistical significance (*p* value from ANOVA is 0.0675), due to stochastic gains and losses of cellular processes such as senescence at the population level. However, the results are informative in showing observable alterations and patterns of the mode expression or trend of breast cancer subtypes that does not necessarily need to be statistically significant. The TN subtype has, on average, lower methylation level than the other two subtypes, suggesting that more genes are activated in TN breast cancers, a more malignant state of breast cancers, than the other subtypes [8, 9]. PCA results showed that TN and luminal cancers can be well separated along the first principle component **(**Fig. 1b, c).

### Prognostic methylation profiles differentiating breast cancer subtypes

There are 2690 differentially methylated CpGs between luminal and TN subtypes (‘Luminal vs TN’, 80.1% of all differentially methylated CpGs, Fig. 1d), 354 between HER2p and TN subtypes (‘HER2p vs. TN’, 10.5% of all differentially methylated CpGs, Fig. 1d). There are 183 hyper-methylated and 130 hypo-methylated CpGs shared between ‘Luminal vs TN’ and ‘HER2p vs. TN’ comparisons, which correspond to 191 differentially methylated genes (Additional file 1: Table S1, S2, S4). Therefore, we obtained 313 CpGs and 191 pDMGs.

**Fig. 2:**
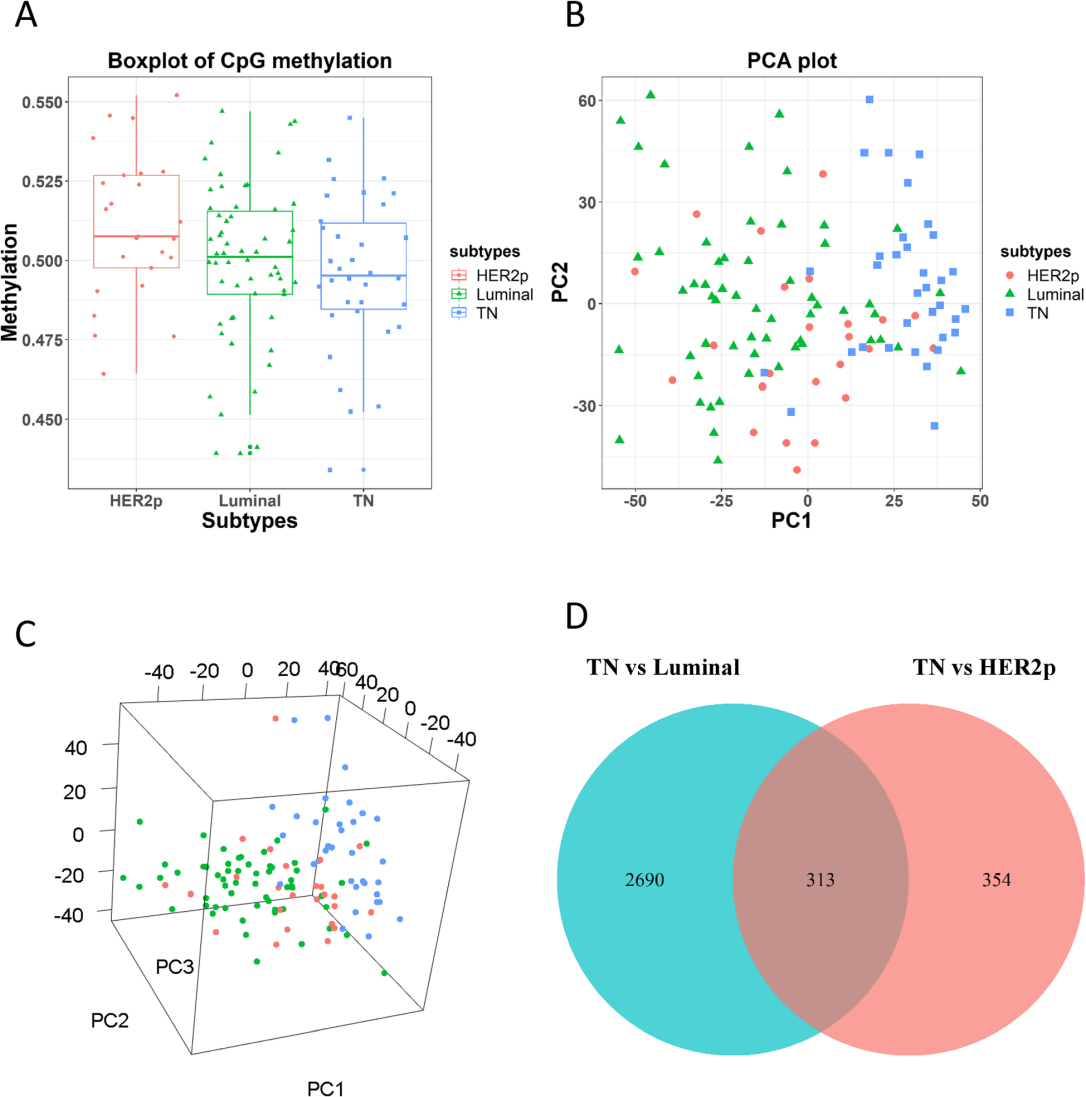
Methylation profiles across breast cancer subtypes in the discovery dataset. (**a**) Methylation of all CpGs across 3 canonical breast cancer subtypes. (**b**) Two-dimentional PCA plot on all CpGs across breast cancer subtypes. (**c**) Three-dimensional PCA plot on all CpGs across breast cancer subtypes. (**d**) Differentially methylated CpGs across different subtype-wise comparisons. The discovery dataset is GSE72251

Applying the pCpGs to the discovery dataset through hierarchical clustering showed 4 distinct patterns across sample subtypes, which correspond to the TN, HER2p and two luminal sample cohorts (Fig. 2a). Purity test revealed that our pCpGs performs better in identifying TN tumors (over 90% purity maximum) than differentiating all the three subtypes (around 80% purity maximum); and the purity reaches the plateau when the number of clusters reached 3 or above in differentiating TN and non-TN tumors, implicating the existence of at least three distinct sample cohorts (excluding HER2) regarding their methylation profiles (Fig. 2b). PCA analysis revealed that the first principle component could distinguish TN and non-TN breast cancers into separate groups, suggesting that the identified pCpGs could capture the primary molecular differences between these subtypes (Fig. 2c). Breast cancer 10-year OS using the pCpGs suggest patients could be stratified into two distinct groups regarding their outcome (*p* = 0.0199, HR = 11.53, Fig. 2d).

### Performance validation of prognostic methylation profiles

**Fig. 3:**
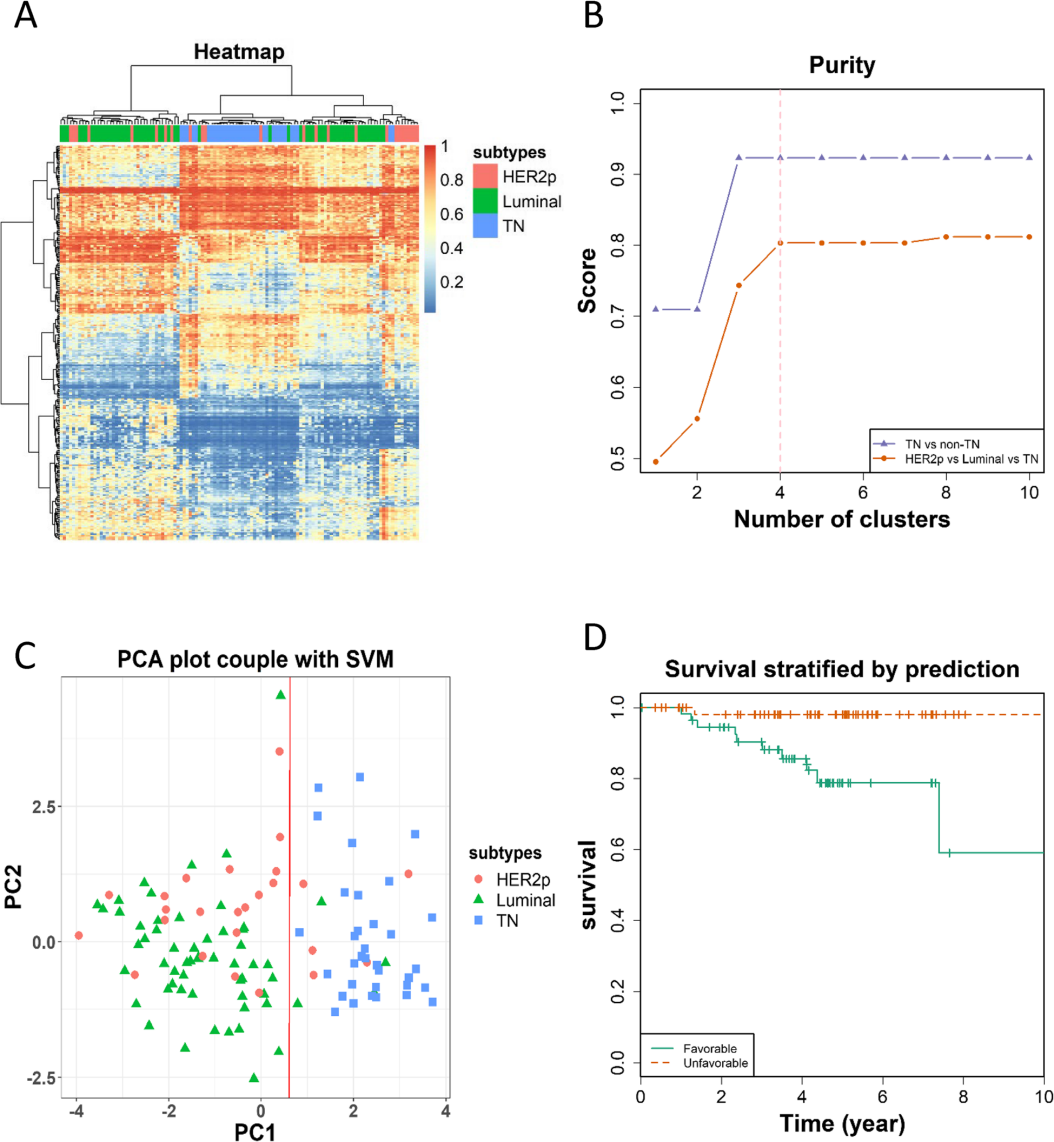
Performance evaluation of pCpGs using the discovery dataset. (**a**) Heatmap showing breast cancer subtypes classified using pCpGs. (**b**) Purity of clusters obtained from hierarchical clustering using pCpGs. (**c**) PCA plot coupled with support vector machine in clustering breast cancer samples based on pCpGs. (**d**) Kaplan Meier survival curves stratified by pCpGs

We used the GSE72245 dataset to validate our pCpGs (Fig. 3a, d, g, j) and TCGA data to validate pDMGs regarding both the methylation (Fig. 3b, e, h, k) and transcriptional profiles (Fig. 3c, f, i, l), with results being consistent with those obtained using the discovery dataset. The alteration directions of methylation and transcriptional profiles are opposite in pDMGs (Additional file 1: Table S3).

**Fig. 4:**
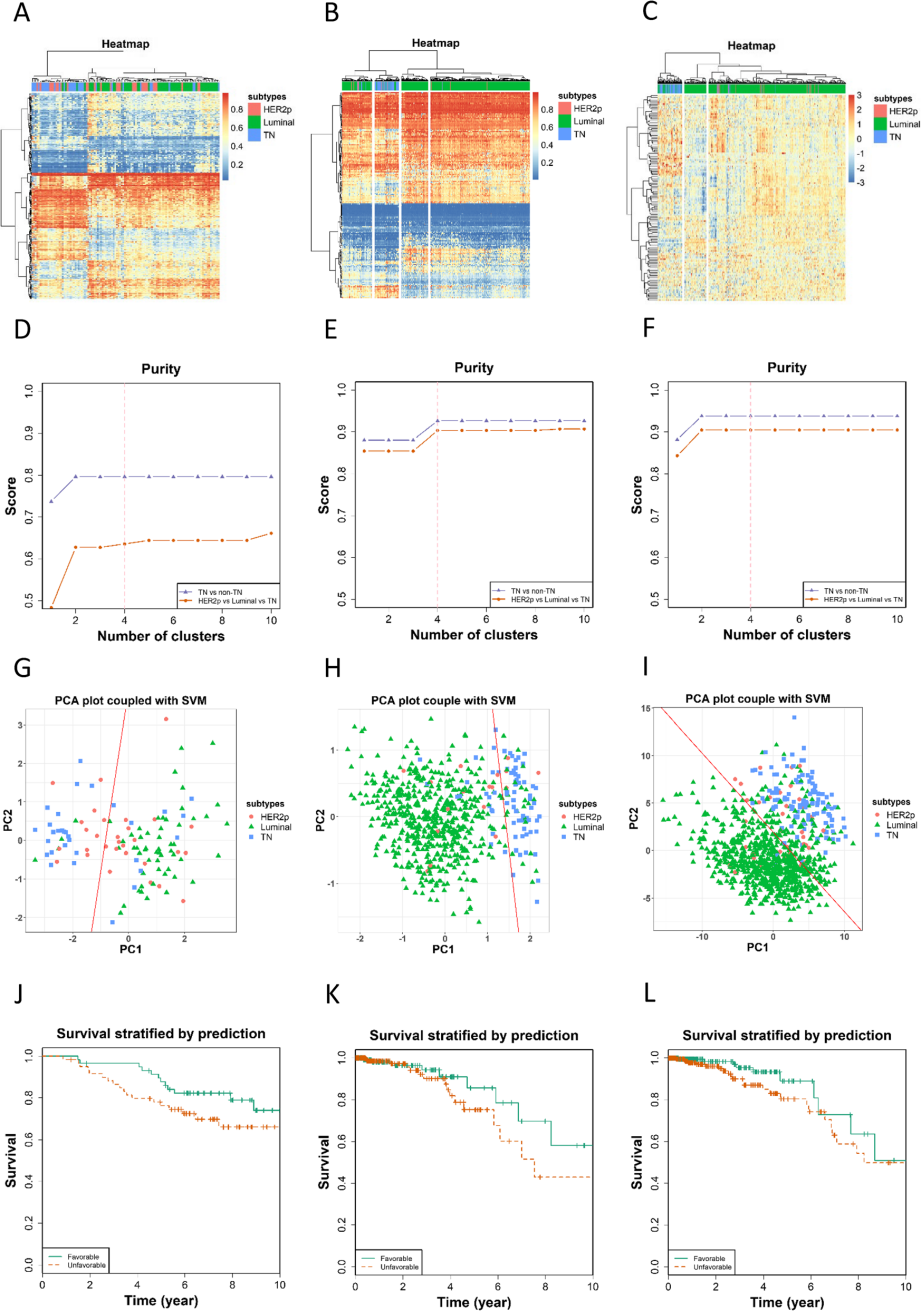
Performance validation of pCpGs and pDMGs using the validation datasets. Heatmaps showing breast cancer subtypes classified using (**a**) pCpGs of GSE72245 on gene methylation, pDMGs of TCGA on (**b**) gene methylation and (**c**) gene expression. Purity of clusters obtained from hierarchical clustering using (**d**) pCpGs of GSE72245 on gene methylation, pDMGs of TCGA on (**e**) gene methylation, and (**f**) gene expression. PCA plot coupled with support vector machine in clustering breast cancer samples using (**g**) pCpGs of GSE72245 on gene methylation, pDMGs of TCGA on (**h**) gene methylation, and (**i**) gene expression. Kaplan Meier survival curves stratified using (**j**) pCpGs of GSE72245 on gene methylation, pDMGs of TCGA on (**k**) gene methylation and (**l**) gene expression

The ROCs constructed from random forest classification using our validation datasets revealed an AUC of 0.88, 0.82, 0.95, respectively, for pCpGs, methylation and gene expression of pDMGs (Fig. 4).

Six genes (including genes encoding ER, PR, HER2 and their transcription factors MYC, FOXA1, MYBL2) were removed from PAM50 (the new panel is named PAM50–6) to exclude their confounding effect on the classifier, as the ground truth was based on tumor classification stratified by ER, PR and HER2. The confusion matrix showed that our pDMGs and the PAM50 each has 563 and 577 samples correctly classified using the TCGA mRNA data (with the F1_nonTN score being 0.97 for pDMGs and PAM50–6; F1_TN score being 0.70 and 0.79, respectively, for pDMGs and PAM50–6) (Table 1).

**Table 1:** The confusion matrix of Random Forest classification using the proposed methylation signature

Signature	Data type	Dataset		TN	nonTN	F1 score		MCC
						F1_TN	F1_nonTN	
pCpGs	Methylation	GSE72245	TN	7	2	0.67	0.90	0.58
			non-TN	5	32			
pDMGs	Methylation	TCGA	TN	11	43	0.29	0.93	0.26
			non-TN	12	384			
pDMGs	mRNA	TCGA	TN	44	25	0.70	0.97	0.67
			non-TN	12	519			
PAM50	mRNA	TCGA	TN	60	9	0.84	0.98	0.82
			non-TN	14	517			
PAM50–6	mRNA	TCGA	TN	55	15	0.79	0.97	0.76
			non-TN	15	515			

The values show the number of consistent and inconsistent samples clustered using each signature panel and identified using immunohistochemistry staining. F1 score that captures both false positives and false negatives is used to assess the classification accuracy. ‘TN’ and ‘non-TN’ are triple negative and non-triple negative breast cancers, respectively. PAM50 and PAM50–6 (with ER, PR, HER2, FOXA1, MYC, MYBL2 excluded) are used as a benchmark. ‘F1_TN’ and ‘F1_nonTN’ each refers to F1 scores in identifying TN and non-TN tumors

### Functional analysis of prognostic methylation genes

**Fig. 5:**
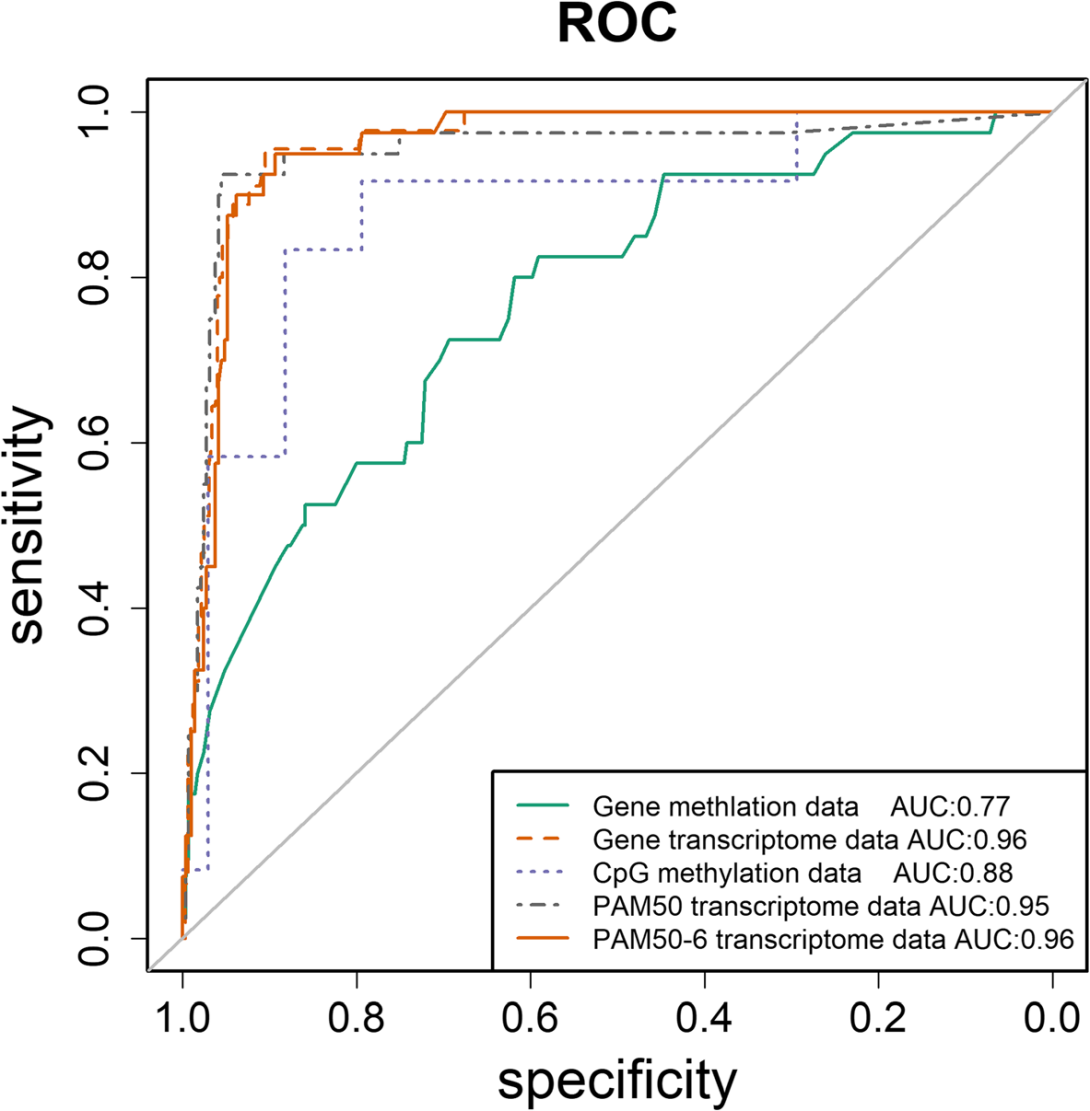
Performance evaluation using RandomForest algorithm. PAM50 was used as the benchmark to evaluate the performance of random forest for learning pCpGs and pDMGs using TCGA data

The 191 pDMGs were enriched in 32 GO terms and 3 KEGG pathways. The top 10 GO terms fell into 3 categories, which are ‘extracellular matrix organization and cell movement’, ‘kinase signaling and cell proliferation’, ‘morphogenesis and cell differentiation’, with ‘extracelluar structure organization’ and ‘extracellular matrix organization’ being the top 2 (Fig. 5a). The 3 top KEGG pathways are ‘focal adhesion’, ‘Wnt signaling pathway’ and ‘Hippo signaling pathway’ (Fig. 5b), which correspond to ‘cell movement’, ‘cell proliferation’ and ‘cell differentiation’ processes, respectively.

**Fig. 6:**
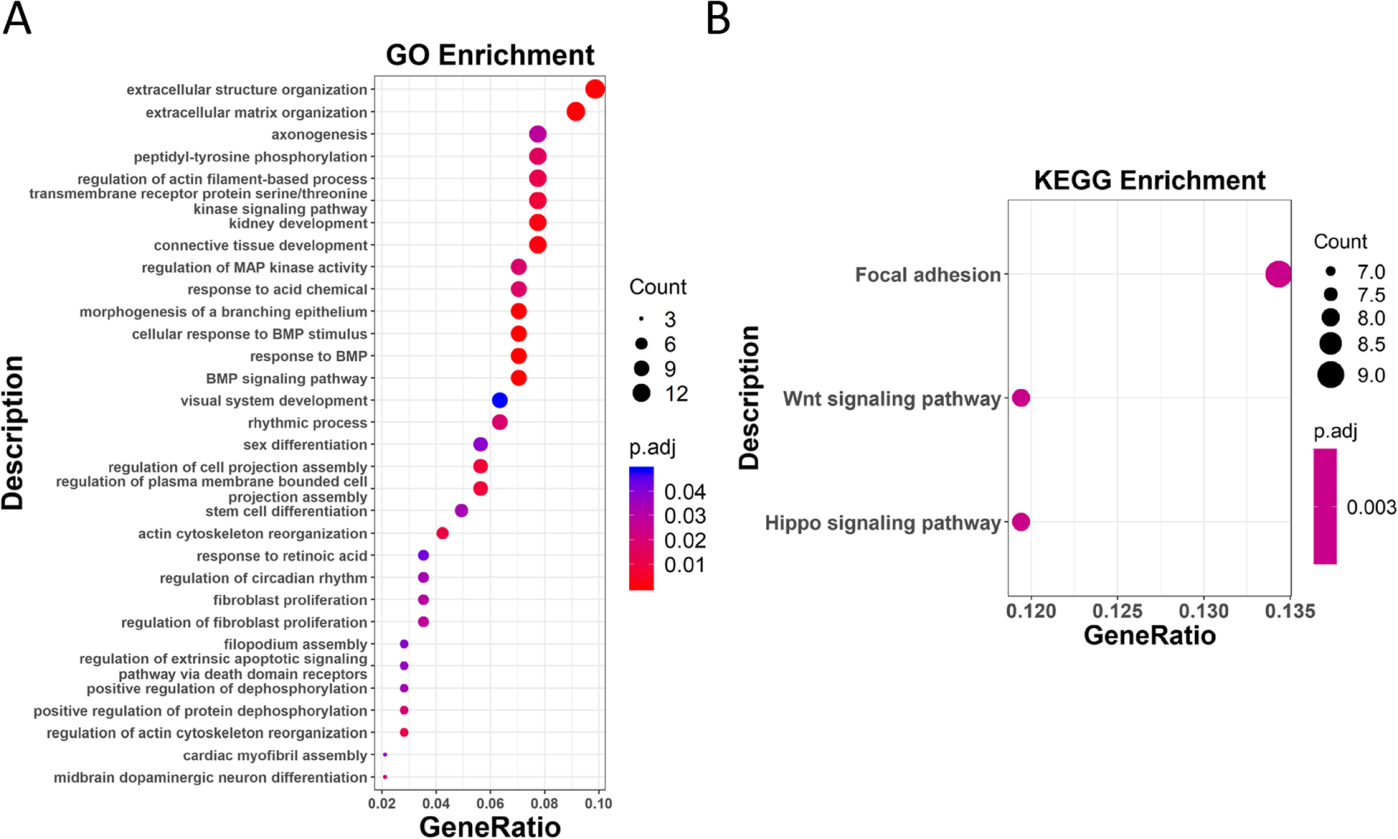
Functional enrichment analysis of pDMGs. (**a**) KEGG pathway analysis of pDMGs. (**b**) GO enrichment of pDMGs. The x-axis shows the GeneRatio (denoted as N_GO_/N_Total_), where N_GO_ is the number of genes from the genes of interest that fell in the targeted GO term, and N_Total_ represents the total number of genes of interest. The dot size represents the number of genes, where the larger the dot size is the greater the number of genes included in the associated GO term. The color denotes the adjusted *p* value, where the more reddish the color is the smaller the *p* value is, and the more bluish the color is the larger the *p* value is. The cutoff between blue and red dots is *p* = 0.025

Among the 191 pDMGs, 7 genes, *MATK*, *IFI35, FAM150B, LBXCOR1*, *WNT10A, ABLIM1* and *CPT1A* were found to be significantly differentially methylated and expressed with opposite clinical associations (Fig. 6). The hypo-methylation and higher expression of *MATK*, *IFI35, FAM150B, LBXCOR1* and *WNT10A* are associated with favorable patient survival, while those of *ABLIM1* and *CPT1A* are associated with poor patient outcome (Table 2).

**Table 2:** Ten years overall survival of breast cancer patients using the 7 pDMGs associated with clinical outcome

Gene	Data Type	Hazard Ratio	P value
ABLIM1	Methylation	0.40	6.91E-03
	Gene expression	2.03	1.23E-02
CPT1A	Methylation	0.35	3.64E-03
	Gene expression	2.45	8.72E-04
FAM150B	Methylation	1.85	4.84E-02
	Gene expression	0.49	1.53E-02
IFI35	Methylation	2.29	4.22E-02
	Gene expression	0.57	3.53E-02
LBXCOR1	Methylation	2.65	8.55E-03
	Gene expression	0.53	2.18E-02
MATK	Methylation	2.12	2.82E-02
	Gene expression	0.44	4.77E-03
WNT10A	Methylation	2.96	1.11E-02
	Gene expression	0.55	3.23E-02

Cox regression analysis was used. Both methylation status and gene expression level were used for patient stratification

## Discussion

We identified 313 pCpGs which correspond to 191 pDMGs capable of distinguishing breast cancer subtypes and especially identifying TN breast cancers. The performance of the 191 pDMGs is similar with that of PAM50–6 in identifying non-TNBCs, which has been clinically used for subtyping and prognosis as BioClassifier™ [10] and ProSigna® [11]; however, as a classifier, pDMGs does not outperform PAM50–6 due to the small size of TNBC cohort. This suggests that these differentially methylated genes could effectively capture the molecular heterogeneity of breast cancers, but the power in identifying small size of tumors may be largely compromised by the restriction that genes need to be under epigenetic regulation in the epigenetic classifier.

Among these 191 pDMGs, 23 are associated with breast cancer outcome and driven by differential methylation status, given the opposite clinical associations and subtype distributions between the methylation and gene expression levels. Out of the 23 pDMGs, 7 have reached statistical significance on clinical associations at both methylation and gene expression levels, which are *MATK, IFI35, FAM150B, LBXCOR1, WNT10A, ABLIM1, CPT1A.*

Though not many, several evidence exist to support the roles of these 7 pDMGs played in carcinogenesis. *MATK* encodes the megakaryocyte-associated tyrosine-protein kinase that can phosphorylate and inactivate the SRC protein, which is one of the 5 markers used in ProEx™Br for breast cancer prognosis, in vitro [12]. The *IFI35* gene is located in the centromeric region and 500 kb away from the *BRCA1* gene in the genome, and suppresses NFkB signaling that plays a promotive role in carcinogenesis [13]. *LBXCOR1* encodes a transcriptional corepressor of *LBX1* and inhibits BMP signaling which predisposes colorectal cancers [14, 15]. *ABLIM1* encodes the actin binding LIM protein 1, whose over-activation promotes tumorigenesis in, e.g., brain and pancreas [16, 17]. *CPT1A* is involved in the fatty acid oxidation pathway [18] and has been proposed as a target of cancers such as nasopharyngeal [19] and prostate [18] carcinomas. It might be possible that malignant cells have accelerated metabolism to meet their up-regulated requirements on biomass production, and targeting *CPT1A* could kill cancers cells through disrupting their fast and efficient fatty acid oxidation. TNBCs have lower *CPT1A* expression than non-TNBCs, suggesting that while accelerated fatty acid metabolism is a characteristic feature of non-TNBCs, the malignancy of TNBCs is driven by other mechanisms such as cell migration and cancer stemness. It was also reported that dietary fat can perturbate genomic structure by reducing DNA methylation at *CPT1A* gene [20], suggesting an over-dose of *CPT1A* expression on high fat dietary exposure that contributes to cancer cell malignancy and warranting our attention to adopting low fat dietary in reducing the risk of developing cancers.

**Fig. 7:**
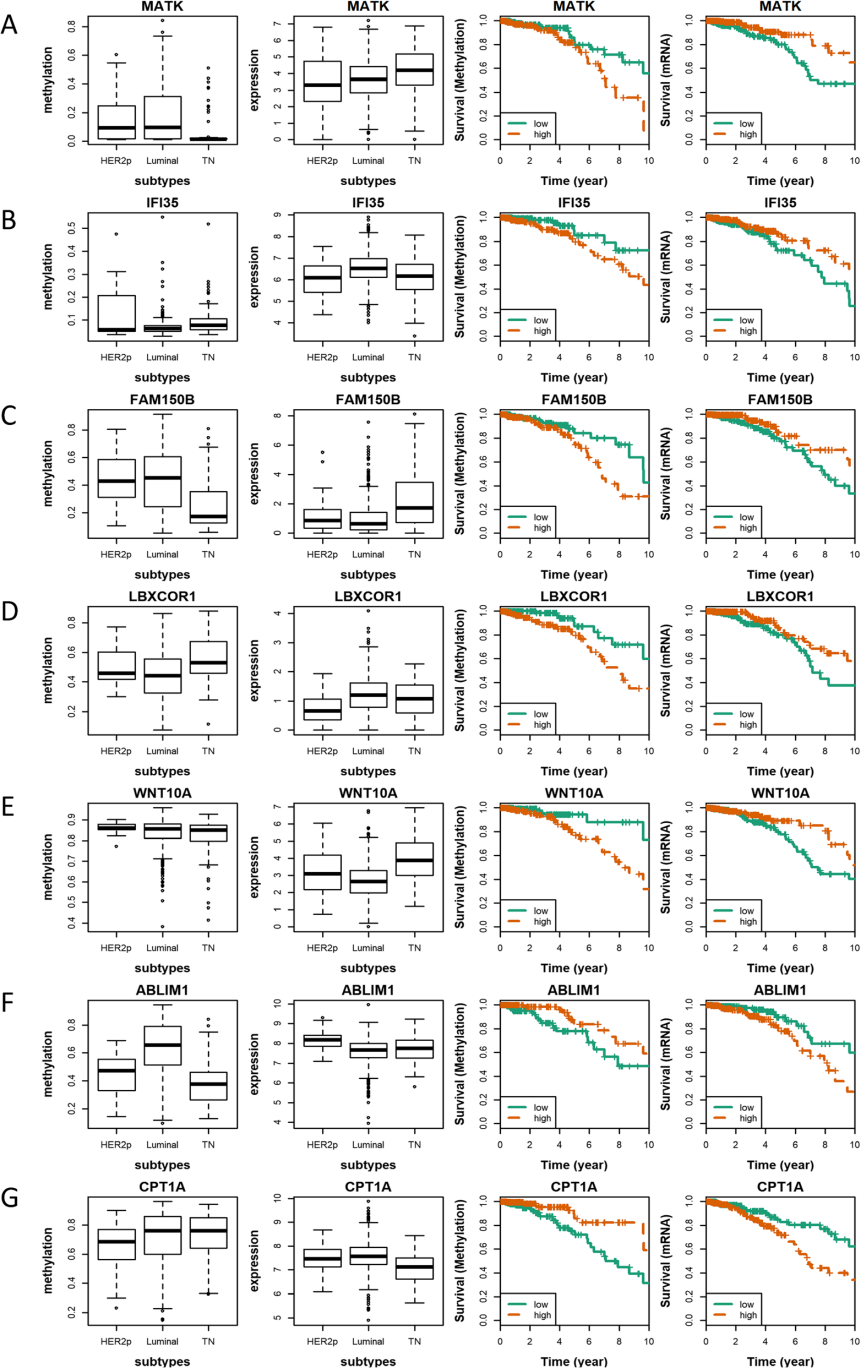
Differentially methylated genes with prognostic values. (**a**) Methylation and gene expression levels, together with the Kaplan Meier plot of each gene at both methylation and gene expression levels are presented for (**a**) MATK, (**b**) IFI35, (**c**) FAM150B, (**d**) LBXCOR1, (**e**) WNT10A, (**f**) ABLIM1, (**g**) CPT1A

Several of these 7 pDMGs may be novel players or have novel roles during carcinogenesis and deserve further investigations. *FAM150B* hyper-methylation was shown to suppress its expression and be associated with poor clinical outcome (Fig. 7c), where direct evidence between *FAM150B* methylation and cancer has not been reported according to our knowledge. *WNT10A* functions as an oncogene in renal cell carcinoma, whose depletion was reported to prevent tumor growth in vitro and in vivo in melanoma [21]; however, it shows tumor suppressive roles in breast cancers in our study which worth further investigations.

The pDMGs identified are largely involved in extracellular structure organization, the regulation of actin filament-based process, transmembrane receptor protein serine/threonine kinase signaling pathway, and connective tissue development, which are all indispensable during cell movement and known to play critical roles in breast cancer progression [22]. These pDMGs are enriched in focal adhesion according to our KEGG pathway analysis (Fig. 5b), which promotes breast cancer initiation and progression once deregulated [23]. MAPK and Wnt pathways are the second most enriched GO terms or pathways of these pDMGs following extracellular structure organization and local adhesion. Both MAPK and Wnt signalings have known connections with carcinogenesis, whose aberration enables cells with uncontrolled proliferation abilities. MAPK and Wnt signalings have cross-talks through TGFβ signaling via a Smad-independent manner [24], and can suppress or promote each other under different circumstances. For example, increased MAPK signaling could down-regulate the Wnt pathway by stabilizing Axin in melanoma, and Wnt signaling activates the MAPK pathway through Ras stabilization in colorectal cancers [25, 26].

## Conclusion

We identified 313 CpGs, corresponding to 191 differentially methylated genes, which capture the molecular differences among breast cancer subtypes with accuracy equivalent to that of PAM50. ‘Cell migration’ as represented by extracellular matrix organization and ‘cell proliferation’ as mediated via MAPK and Wnt signalings were identified as primary factors stratifying breast cancer subtypes that are modulated via aberrant methylations. Our study provides DNA methylation profiles with prognostic values and clinical translation potential, and offers novel insights on the driving force orchestrating breast cancer heterogeneity from the epigenetic perspective.

## Material and methods

### Data

The GEO dataset, GSE72251, performed using the Illumina Infinium Human Methylation Beadchip (450 k array), was retrieved from the NCBI Gene Expression Omnibus (GEO) database [27] and used as the discovery dataset, which is consisted of 119 breast cancer samples and 415,080 CpGs.

The GEO dataset, GSE72245, was retrieved and used as one validation dataset, which encompasses 118 samples and 415,080 CpGs.

Both GSE72251 and GSE72245 were preprocessed by removing high-detection *p*-values, SNP-containing, cross-reactive and heterochromosomic probes (which were replaced by ‘null’) and both datasets were normalized using the peak-based approach. Methylation and mRNA data as well as clinical information from The Cancer Genome Atlas (TCGA) [28] were downloaded from the cBioportal [29] and used as another validation dataset. This data is comprised of 550 samples and 16,474 genes. Based on the assumption that DNA methylation is a common epigenetic signaling tool that cells use to lock genes in the ‘off’ state [30], only the CpG probe showing the strongest negative correlation with gene expression was kept and used as the methylation probe of the gene when multiple probes existed to target one single gene. Besides, as pCpGs with gene regulatory roles typically occur in the promoter region of the targeted gene [30], it is unlikely to select a CpG that is functionally irrelevant to the gene in question using this function-based approach, e.g., if a CpG happened to be located in the 3’UTR of gene A and in the promoter region of gene B, its association could only be possibly found with gene B but not with gene A.

All samples were classified into triple negative (TN), HER2 positive (HER2p) and luminal subtypes according to estrogen receptor (ER), progestogen receptor (PR) and human epithelial receptor 2 (HER2) immunohistochemistry status (i.e., HER2p = ER-PR-HER2+, luminal = ER + |PR+, TN = ER-PR-HER2-). Although luminal cancers can be further divided to the A and B subtypes, they share similar molecular patterns and are considered as one large class in many studies [31] including this paper.

### Differential methylation analysis

Differential methylation analysis was performed based on student T test and Bayes theorem using the ‘limma’ [32] package from the Bioconductor [33] package. Based on the empirical Bayes method (the ‘eBayes’ function), CpG sites specifically hypo-methylated or hyper-methylated in TN breast cancers were ranked in the order of the statistical significance of methylation difference, where the Benjamin-Hochberg adjusted *P*-values < 0.01 was used as the significance threshold.

### Survival analysis

The 10-year breast cancer overall survival (OS) analysis of the selected differentially methylated CpGs or genes (DMGs) was performed using the methylation profiles and the clinical data. The analysis was conducted using the Cox proportional hazards model, with the logrank *p*-value less than 0.01 being considered statistically significant. We defined CpGs with prognostic significance as pCpG and genes where pCpG reside in as pDMGs. To interrogate the prognostic value of a panel of methylation sites, we implemented a multi-methylation survival analysis where each methylation site was assigned with 1 (favorable) or 0 (unfavorable), defined as the prognostic score, according to the univariate survival analysis; and an averaged prognostic score of each sample was calculated by averaging the prognostic scores over all methylation sites for each sample, and used for sample stratification in the survival analysis.

### Hierarchical clustering and purity analysis

Hierarchical clustering, an unsupervised machine learning approach, was performed using the Euclidean or pair-wise sample correlation (1-r, where ‘r’ represents the correlation) distance and the Ward linkage [34]. The subtyping performance of pDMGs was assessed by the purity statistics at cutoffs ranged from 1 to 10. The purity of each cluster was computed by assigning it to the most represented breast cancer subtype by its encompassed nodes following the calculation of the fraction of nodes with correct assignment in each cluster.

### Principle component analysis and support vector machine classification

Principle component analysis (PCA) was conducted using the `prcomp` function from the ‘base’ package in R. Support vector machine (SVM) was used to classify samples projected by PCA via the ‘svm’ function from the ‘e1071’ package in R.

### Random forest and receiver operating characteristic curve construction

Random forest classification was conducted using the ‘randomForest’ function from ‘randomForest’ package in R. The number of nodes in a tree was determined through iterations from 1 to ‘n-1’ where ‘n’ represents the sample size, and the one with the minimum error was picked. Receiver operating characteristic curve (ROC) and the area under the curve (AUC) were computed using the ‘roc’ function from the ‘pROC’ package in R to assess the clustering accuracy. To take both false positives and false negatives into account in the assessment, F1 score was calculated as below:

$$ \mathrm{F}1=2\times \frac{Recall\times Precision}{Recall+ Precision} $$

where $$ \mathrm{Precision}=\frac{TP}{TP+ FP} $$ and $$ \mathrm{Recall}=\frac{TP}{TP+ FN} $$

### Functional analysis

Functional enrichment analysis was performed based on Gene Ontology (GO) [35] and Kyoto Encyclopedia of Genes and Genomes database (KEGG [36]) using the R package ‘clusterProfiler’ [37]. Fisher’s exact test was utilized to measure the significance of GO terms and biological pathways. The *p*-values were adjusted using Benjamini-Hochberg false discovery rate (FDR), and *p* < 0.01 was used as the threshold to assess the statistical significance of each test [38]. The overall workflow is demonstrated in Fig. 7.

## Supplementary information

**Additional file 1: Table S1.** Annotations of prognostic CpGs. **Table S2.** Annotations of prognostic DMGs. **Table S3.** Correlation between the methylation and expression of pDMGs. **Table S4.** Learning matrix of the 313 CpG (with gene attribution) and outcome variables.

## Data Availability

GSE72251 and GSE72245 datasets were retrieved from the NCBI Gene Expression Omnibus (GEO) database [27]. Methylation and mRNA data as well as clinical information of The Cancer Genome Atlas (TCGA) [28] dataset were downloaded from the cBioportal (http://download.cbioportal.org/brca_tcga.tar.gz) [29].

## Abbreviations

AUC: Area under the curve
DMGs: Differentially methylated genes
ER: Estrogen receptor
FDR: False discovery rate
GEO: Gene expression omnibus
GO: Gene ontology
HER2: Epithelial receptor 2
HER2p: HER2 positive
KEGG: Kyoto encyclopedia of genes and genomes database
OS: Overall survival
PCA: Principle component analysis
pCpG: Prognostic differentially methylated CpG
pDMG: Prognostic differentially methylated gene
PR: Progestogen receptor
ROC: Receiver operating characteristic curve
SVM: Support vector machine
TCGA: The Cancer Genome Atlas
TN: Triple negative
